# The value of apparent diffusion coefficient values in predicting Gleason grading of low to intermediate-risk prostate cancer

**DOI:** 10.1186/s13244-024-01684-x

**Published:** 2024-06-09

**Authors:** Xu Yan, Ke Ma, Li Zhu, Yiqi Pan, Yuting Wang, Jiong Shi, Xiaoli Mai

**Affiliations:** 1 Department of Radiology, Nanjing Drum Tower Hospital, Affiliated Hospital of Medical School, Nanjing University, Nanjing, 210008 China; 2 Department of Radiology, Nanjing Drum Tower Hospital Clinical College of Xuzhou Medical University, Nanjing, 210008 China; 3 Department of Radiology, Nanjing Drum Tower Hospital Clinical College of Nanjing Medical University, Nanjing, 210008 China; 4 Department of Pathology, Nanjing Drum Tower Hospital, Affiliated Hospital of Medical School, Nanjing University, Nanjing, 210008 China

**Keywords:** Prostate cancer, Magnetic resonance imaging, Apparent diffusion coefficient, Gleason score

## Abstract

**Objectives:**

To investigate the diagnostic performance of the apparent diffusion coefficient (ADC) for low to intermediate-risk prostate cancer (PCa), as well as its correlation with the prognostic Gleason score (GS).

**Materials and methods:**

Retrospective analysis of MRI images and relevant clinical data from patients with prostate disease. The differences in ADC between different GS groups were compared, and the efficacy of ADC in PCa diagnosis were analyzed. Furthermore, the diagnostic performance of the mean ADC (ADC_mean_) and minimum ADC (ADC_min_) values was compared.

**Results:**

There were 1414 patients with 1631 lesions. In terms of GS, both ADC_min_ and ADC_mean_ values of the GS 4 + 3 group were significantly lower than those of the GS 3 + 4 group, GS 3 + 3 group, and the benign group, with all differences being statistically significant (*p* < 0.01). The AUC values for diagnosing PCa based on ADC_min_ and ADC_mean_ were 0.914 and 0.944, respectively. The corresponding diagnostic thresholds were 0.703 × 10^−^^3^ mm^2^/s for ADC_min_ and 0.927 × 10^−3^ mm^2^/s for ADC_mean_. The magnitudes of ADC_min_ and ADC_mean_ values exhibited a negative correlation with GS values (*ρ* = −0.750, *p* < 0.001; *ρ* = −0.752, *p* < 0.001).

**Conclusions:**

ADC values demonstrate an inverse relationship with the invasiveness of PCa, indicating that higher invasiveness is associated with lower ADC values. Additionally, ADC values exhibit high diagnostic potential, sensitivity, and specificity for distinguishing between GS 3 + 4 and GS 4 + 3 lesions. Moreover, the diagnostic value of ADC_mean_ is even more significant, highlighting its crucial role in the diagnosis of low to intermediate-risk PCa.

**Critical relevance statement:**

ADC values are a valuable tool for distinguishing different levels of aggressiveness in PCa. They help in the preoperative assessment of the biological characteristics of PCa, allowing clinicians to develop personalized treatment strategies, effectively mitigating the risk of unnecessary interventions.

**Key Points:**

- The preoperative GS is crucial for planning the clinical treatment of PCa.
- The invasiveness of PCa is inversely correlated with ADC values.
- ADC values play a crucial role in the accurate preoperative evaluation of low to intermediate-risk PCa, thus aiding clinicians in developing tailored treatment plans.

**Graphical Abstract:**

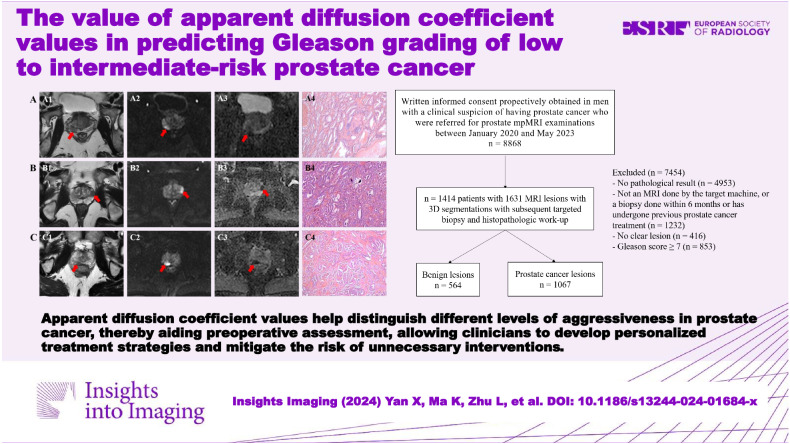

## Introduction

Prostate cancer (PCa), ranking second only to lung cancer in terms of incidence, occupies the second position among malignancies affecting males worldwide [1]. In China, it stands as the sixth most prevalent cancer in the male population and is characterized by a consistent upward trajectory [2]. PCa exhibits a pronounced proclivity for bone metastasis [3], leading to the unfortunate scenario where many patients miss the optimal window for treatment upon initial diagnosis, resulting in compromised prognoses. The accurate grading and staging of PCa intricately shape the selection of clinical interventions and directly impact the disease’s anticipated course.

In accordance with the guidelines delineated by the European Association of Urology, the current gold standard for diagnosing PCa is achieved through ultrasound-guided prostate biopsy [4]. However, the invasive nature of biopsy procedures engenders a cascade of potential complications. Recent years have witnessed the ascendancy of non-invasive imaging methodologies that adeptly prognosticate the gradation and extent of PCa.

With the evolution of multiparametric magnetic resonance imaging (mpMRI) technology, a consortium of directives now advises that all men with suspected PCa undergo mpMRI assessments before biopsy [5, 6]. These evaluations encompass a suite of imaging sequences, encompassing T_1_-weighted imaging (T_1_WI), T_2_-weighted imaging (T_2_WI), diffusion-weighted imaging (DWI), and dynamic contrast enhancement. To standardize the acquisition, interpretation, and reporting of mpMRI findings, the collaborative efforts of the European Society of Urogenital Radiology, the American College of Radiology, and the Ad Me Tech Foundation culminated in the release of prostate imaging and reporting data system version 2.1 (PI-RADS V2.1) in 2019 [7]. This version confers more nuanced criteria for evaluating prostate lesions, thereby endeavoring to reduce interpretative variability among medical practitioners.

A central limitation of PI-RADS lies in its inherent subjectivity when assessing the risk and dimensions of lesions, thus falling short in its ability to reliably prognosticate disease outcomes. In contrast, the apparent diffusion coefficient (ADC) value offers a quantitative metric, imbuing the assessment process with a degree of objectivity. This quality, to a certain extent, addresses the foregoing limitation. This present study employs DWI techniques to scrutinize the diagnostic efficacy of ADC values concerning low to intermediate-risk PCa. Furthermore, it explores the correlation between ADC values and the Gleason score (GS), an integral diagnostic parameter. Additionally, an evaluation is conducted to juxtapose the diagnostic potency of the mean apparent diffusion coefficient (ADC_mean_) and the minimum apparent diffusion coefficient (ADC_min_) in appraising prostate lesions.

## Materials and methods

### Ethics statement

This study was a retrospective study, we applied to exempt patients from informed consent, and it was supervised and approved by the institutional review board (Ethics Committee of Nanjing Drum Tower Hospital, Nanjing, China; No. 2023-243-01).

### Patients

**Fig. 1:**
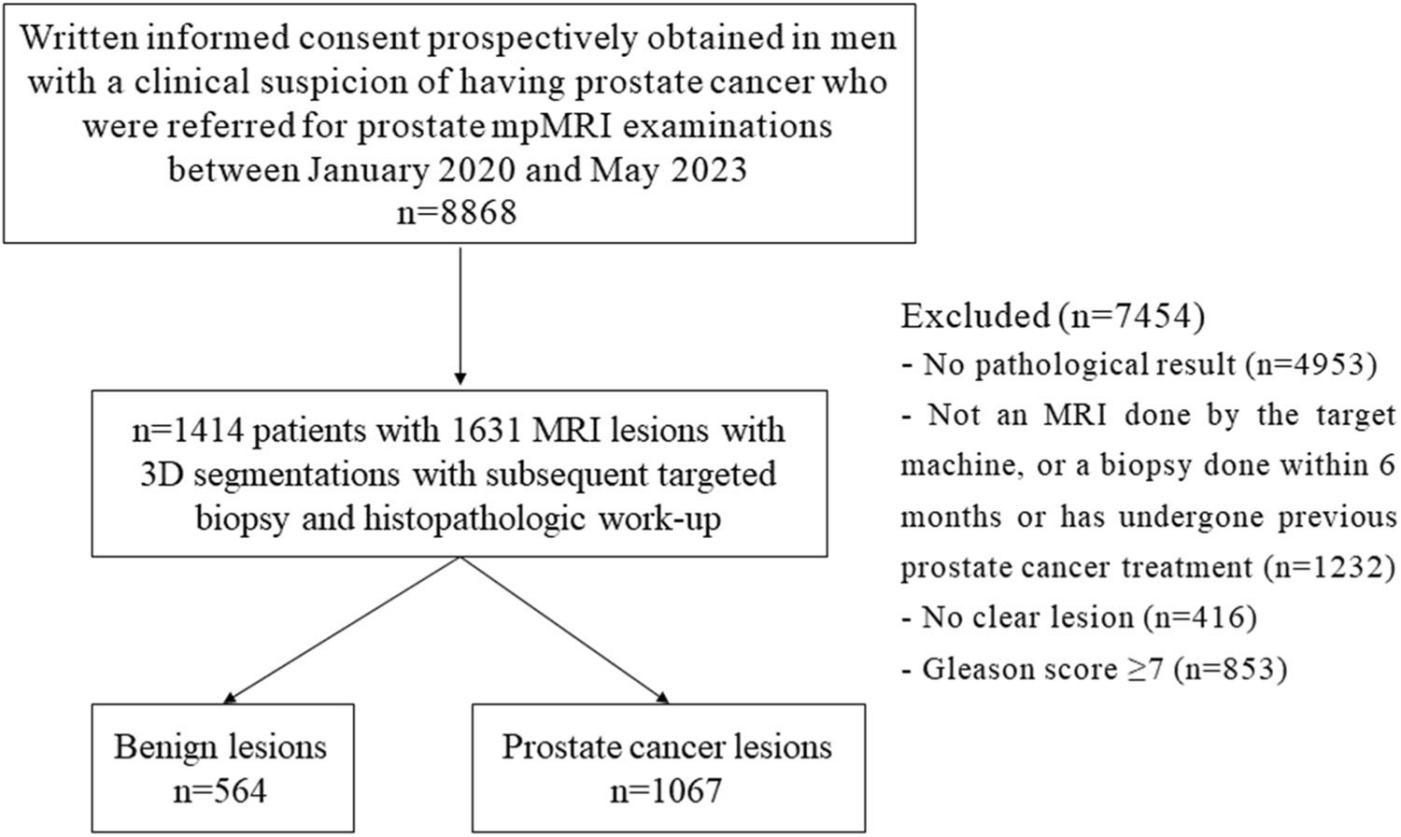
Flowchart of patient inclusion into the study

During the period spanning from January 2020 to May 2023, a comprehensive dataset was curated encompassing 8868 patients who sought medical attention at our institution and underwent prostate MRI evaluations. Patients typically underwent evaluation due to elevated levels of prostate-specific antigen (PSA) or the presentation of symptoms such as urinary difficulties and hematuria. The specific inclusion and exclusion criteria are shown in Fig. 1.

### MRI data acquisition

Utilizing a 3-T MRI scanner (United Imaging, uMR770, Shanghai, China), we harnessed the power of dual-source parallel radiofrequency pulse excitation technology. This advanced system seamlessly integrated a 32-channel phased-array surface coil for the precise acquisition of signals. The meticulously chosen scanning sequences and parameters were meticulously designed as follows: T_1_WI—TR/TE of 612 ms/9.36 ms, T_2_WI—TR/TE of 3000 ms/135.36 ms, Fat-suppressed T_2_WI—TR/TE of 4000 ms/104.78 ms, DWI—TR/TE of 5000 ms/80 ms, *b* = 50/800/1500 sec/mm². The comprehensive scanning procedure was conducted over an approximate duration of 18 minutes.

### Image analysis

The quantification of ADC values within the images was executed using the institutional picture archiving and communication systems (PACS). This robust system serves as a repository for medical images and data.

The meticulous task of image analysis was undertaken by two genitourinary radiologists with years of experience in prostate mpMRI interpretation, and in instances of divergent viewpoints, a harmonized consensus was attained through thorough deliberation. Correlating with the pathology results from biopsy or definitive surgical excision, delineate the region of interest (ROI) on MRI images. Aim for meticulous outlining to encompass the utmost extent of the lesion, while avoiding measurement interference from areas such as the urethra, hemorrhage, necrosis, cystic transformations, and vascular structures. Conduct measurements across all dimensions of the lesion, documenting the minimum value and calculating the average. When measuring, each lesion contains at least two layers.

### Histopathologic analysis

Employing pathological findings as the benchmark of accuracy, we employed the GS system (a 5-tier scale with scores ranging up to 10) to stratify tumors based on their histological grade. Benign lesions (BL) underwent assessment through biopsy-derived pathological results, employing a technique known as transperineal prostate biopsy guided by ultrasound and magnetic resonance fusion. Malignant lesions, on the other hand, were evaluated utilizing histopathology garnered from surgically excised specimens, predominantly originating from radical prostatectomy procedures.

**Fig. 2:**
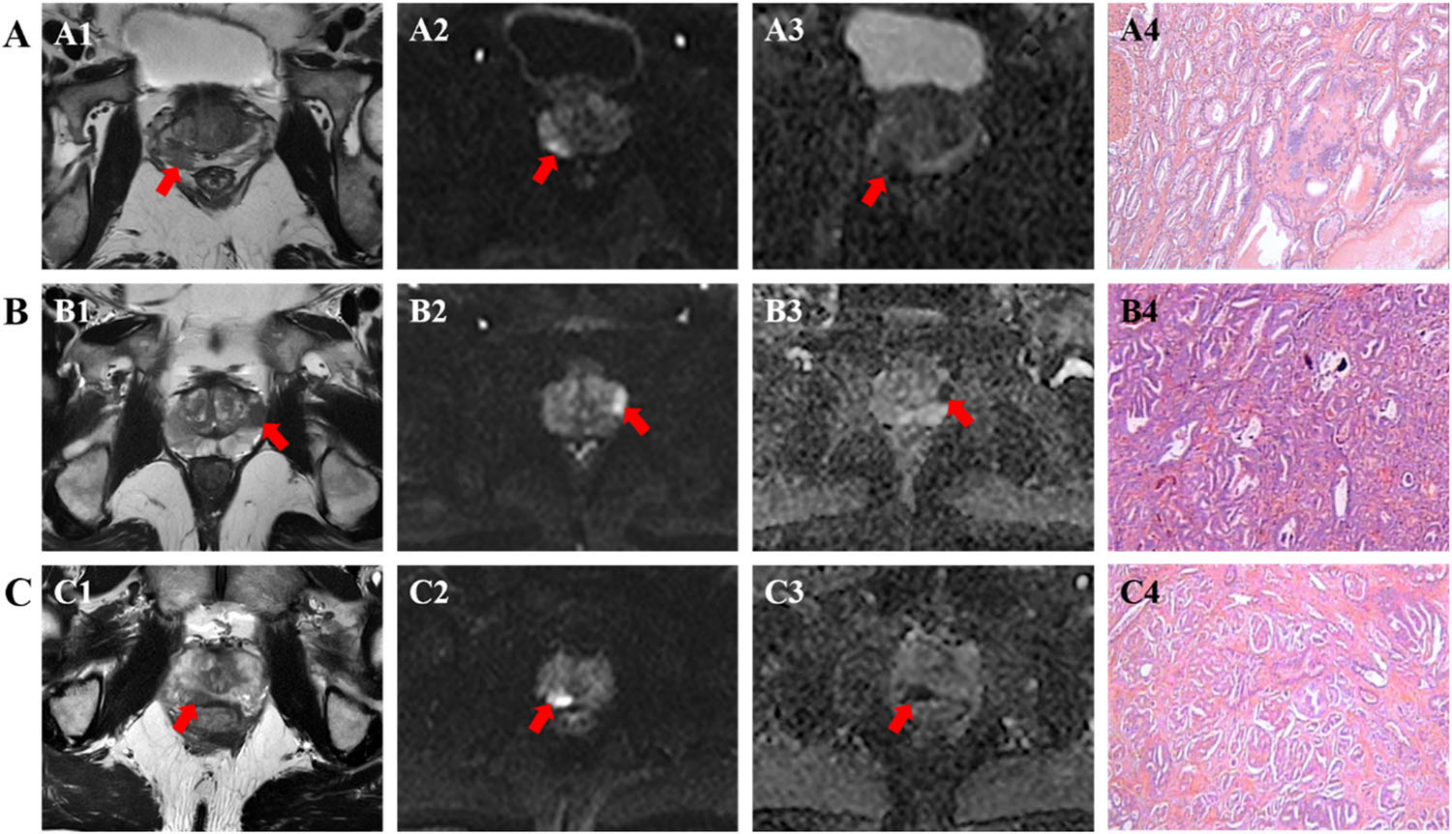
T_2_WI shows low signal (A1, B1, C1, red arrows), DWI shows high signal (A2, B2, C2, red arrows) and ADC shows low signal (A3, B3, C3, red arrows) in peripheral zone. **A** A 68-year-old man, PSA = 11 ng/mL, ADC_min_ = 0.576 × 10^−3^ mm^2^/s (*b* = 800), ADC_mean_ = 0.984 × 10^−^^3^ mm^2^/s (*b* = 800); GS 3 + 3 (HE × 40) (A4). **B** 67-year-old man, PSA = 7.68 ng/mL, ADC_min_ = 0.411 × 10^−^^3^ mm^2^/s (*b* = 800), ADC_mean_ = 0.779 × 10^−^^3^ mm^2^/s (*b* = 800); GS 3 + 4 (HE × 40) (B4). **C** A 72-year-old man, PSA = 6.4 ng/mL, ADC_min_ = 0.071 × 10^−3^ mm^2^/s (*b* = 800), ADC_mean_ = 0.748 × 10^−^^3^ mm^2^/s (*b* = 800); GS 4 + 3 (HE × 40) (C4)

The conclusive diagnostic outcomes distinctly categorized lesions into either benign or malignant entities. Within the domain of BL, conditions encompassed prostate hyperplasia, prostatitis, and unaltered prostate tissue. Malignant lesions were further stratified into GS 3 + 3, GS 3 + 4, GS 4 + 3 (as visually represented in Fig. 2), and GS ≥ 4 + 4. In alignment with internationally acknowledged PCa grading conventions [6], GS 3 + 3 was classified as indicative of low-risk PCa, whereas both GS 3 + 4 and GS 4 + 3 denoted intermediate-risk PCa. Notably, GS ≥ 4 + 4 was unequivocally associated with high-risk PCa status.

### Statistical analysis

Data analysis was conducted utilizing the statistical software GraphPad Prism (Version 8.0, GraphPad Software, San Diego, California, USA) and SPSS (Version 26.0, IBM, Armonk, New York). Employing pathological results as the reference standard, the diagnostic efficacy of ADC values for PCa was assessed. The evaluation commenced by analyzing lesions without stratification by prostate regions, followed by a more refined investigation that differentiated lesions within specific prostate zones, encompassing the peripheral and transition zones. The normality of data distribution was verified, and when met, independent samples t-tests were executed, with results presented as mean ± standard deviation ($\bar{{{{{{\rm{x}}}}}}}$ ± s). In cases where the assumption of normality was not satisfied, non-parametric tests were applied, and results were conveyed as median (interquartile range) (M (P25, P75)).

To assess the diagnostic performance, receiver operating characteristic (ROC) curve analysis was employed to determine ADC diagnostic thresholds for PCa diagnosis and differentiation of distinct Gleason grades. This procedure generated the corresponding area under the curve (AUC), enabling the calculation of sensitivity, specificity, and Youden’s index. Further analyses encompassed the comparison of ADC value discrepancies between benign and malignant lesions, as well as the examination of ADC value variations across diverse Gleason grades. Spearman’s correlation analysis was employed to ascertain the connection between ADC values and GS. Notably, a significance level of *p* < 0.05 was deemed indicative of statistically significant disparities.

## Results

### Clinical data

The study ultimately enrolled 1414 participants, whose ages spanned from 42 to 91 years. Of this total, PSA testing was conducted for 954 patients, while 460 patients either refrained from PSA testing or their PSA results were omitted from the analysis. The distribution of PSA levels is presented in Table 1. This nuanced dataset revealed a distinctive trend wherein higher PSA levels correlated with an augmented likelihood of malignancy.

**Table 1:** Proportion of benign and malignant cases at various PSA levels

PSA (ng/mL)	BL (%)	PCa (%)
< 4	53 (80.30)	13 (19.70)
4–10	262 (53.69)	226 (46.31)
10.01–20	135 (46.08)	158 (53.92)
> 20	37 (34.58)	70 (65.42)

### Pathological results

Following a meticulous selection process, 1414 patients were ultimately included in the study, encompassing a total of 1631 lesions, spinning an age range from 42 to 91 years. The distribution of lesions among varied GS is presented in Table 2. Notably, certain lesions lacking precise allocation within distinct prostate zones were judiciously excluded. Ultimately, 729 cases (56.29%, 42–85 years) were located in the peripheral zone (PZ), while 566 cases (43.71%, 49–91 years) were located in the transitional zone (TZ) (Supplementary Table 1).

**Table 2:** Dispersion of lesions among varied GS

Group	Number	Ratio (%)	Age (years)
Lesion	1631	–	42–91
BL	564	34.58	45–91
PCa	1067	65.42	42–86
GS 3 + 3	210	12.88	49–86
GS 3 + 4	575	35.25	42–85
GS 4 + 3	282	17.29	49–85

### Application value of ADC values in different risk stratifications of PCa

**Fig. 3:**
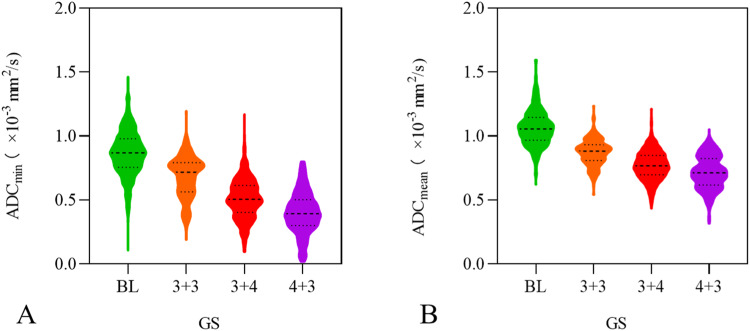
Distribution plots of ADC_min_ (**A**) and ADC_mean_ (**B**) among various GS groups

The distribution patterns of ADC values among distinct cohorts are detailed in Table 3 and Fig. 3A, B. The ascending order of ADC values, from highest to lowest, across diverse patient groups is: BL group, GS 3 + 3 group, GS 3 + 4 group, GS 4 + 3 group. Notably, there are significant disparities in ADC values observed among these distinct groupings (*p* < 0.01). Specifically, the ADC_min_ and ADC_mean_ medians of the GS 4 + 3 group are markedly lower than those of both the GS 3 + 3 and GS 3 + 4 groups, exhibiting statistical significance (*p* < 0.01). Furthermore, meticulous pairwise comparisons between groups unveil statistically significant deviations in ADC values. This includes discrepancies between the benign group and the PCa group, as well as among varying GSs (*p* < 0.01). The discernment of ADC values proves particularly salient in the context of distinguishing distinct Gleason grading cohorts, as evidenced by the results elucidated in Table 4.

**Table 3:** Dispersion of median ADC_min_ and ADC_mean_ values within each respective group

Group	Number (%)	ADC_min_ (× 10^–^^3^ mm^2^/s, *b* = 800)	ADC_mean_ (× 10^–^^3^ mm^2^/s, *b* = 800)	Z value	*p* value
BL	564 (34.58)	0.869 (0.755, 0.980)	1.056 (0.967, 1.146)	–18.454	< 0.001
PCa	1067 (65.42)	0.507 (0.382, 0.645)	0.779 (0.690, 0.867)	–29.923	< 0.001
GS 3 + 3	210 (12.88)	0.718 (0.565, 0.792)	0.882 (0.809, 0.933)	–12.284	< 0.001
GS 3 + 4	575 (35.25)	0.505 (0.404, 0.614)	0.768 (0.698, 0.850)	–24.404	< 0.001
GS 4 + 3	282 (17.29)	0.394 (0.301, 0.503)	0.713 (0.617, 0.825)	–17.728	< 0.001

**Table 4:** Association between ADC and distinct GS groups concerning PCa

	Group	ADC (× 10^–^^3^ mm^2^/s, *b* = 800)	Sensitivity (%)	Specificity (%)	Youden index (%)	AUC
**ADC**_**min**_	PCa	0.703	84	85	68	0.914
	GS 3 + 3	0.815	82	63	46	0.783
	GS 3 + 4	0.649	82	64	46	0.782
	GS 4 + 3	0.464	67	64	31	0.687
**ADC**_**mean**_	PCa	0.927	89	86	75	0.944
	GS 3 + 3	0.986	89	72	61	0.878
	GS 3 + 4	0.809	63	72	40	0.749
	GS 4 + 3	0.695	45	76	21	0.623

**Fig. 4:**
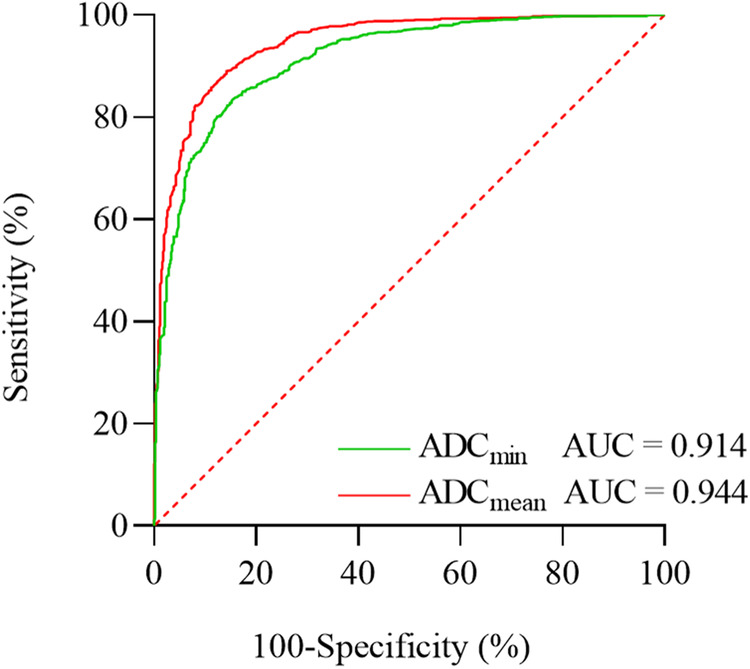
ROC curves for the diagnosis of PCa using ADC_mean_ and ADC_min_

In the diagnostic realm, when employing mpMRI to discern PCa across diverse grades, the ADC_mean_ metric exhibits superior sensitivity, specificity, Youden index, and AUC, compared to the ADC_min_ metric. Such enhanced diagnostic attributes of ADC_mean_ remain consistent throughout the comparison of diagnostic prowess across different Gleason-graded PCa cases, as illustrated in Fig. 4.

### The diagnostic significance of ADC values across diverse prostate regions and risk classification cohorts

Irrespective of whether it pertains to the peripheral or transition zone, both ADC_min_ and ADC_mean_ values of the malignancy group exhibit a significant reduction compared to the benign counterpart, showcasing statistically meaningful distinctions among the groups (*p* < 0.01) (Supplemental Table 2). In a comparative analysis, it is noteworthy that while the ADC_min_ values in the malignancy group are relatively higher within the PZ, and the ADC_mean_ values are comparably higher within the transition zone of the malignancy group, these discrepancies lack statistical significance (*p* > 0.05). From these findings, it becomes evident that in the realm of diagnosing varying Gleason-graded PCa within the peripheral and transition zones, the diagnostic performance of ADC_mean_ remains superior to that of ADC_min_.

### Relationship between ADC values and the Gleason grading system

The Spearman rank correlation analysis yields significant findings, demonstrating a negative correlation between PCa ADC_min_ and GS (*ρ*_s_ = –0.764, *p* < 0.001), as well as between ADC_mean_ and GS (*ρ*_s_ = –0.845, *p* < 0.001). Notably, with escalating GSs, there is a consistent and discernible decline in ADC values.

## Discussion

Our study demonstrates that ADC **v**alues can effectively differentiate between different invasive lesions, which is beneficial for the preoperative evaluation of PCa.

This research elucidates that with the elevation of serum PSA levels, the risk of PCa increases progressively [8]. Currently, clinical guidelines advocate for early serum PSA testing among individuals at high risk of PCa, including men aged over 50, those over 45 with a familial history of PCa, men over 40 with PSA > 1 ng/mL, and those over 40 carrying BRCA2 gene mutations [9]. Although conventional norms designate serum PSA levels under 4 ng/mL as within the normal range, it’s important to recognize that PSA levels within this interval do not comprehensively rule out the potential presence of PCa. Accurate diagnosis necessitates a comprehensive approach involving digital rectal examinations, biopsies, and MRI scans, aligning with insights from various studies and clinical guidelines [9, 10]. For PSA > 10 ng/mL, prompt prostate biopsy is recommended. Conversely, in the PSA “gray zone” spanning 4 to 10 ng/mL, an intricate assessment involving the free/total PSA ratio or prostate-specific antigen density is deemed prudent [11].

Presently, the definitive preoperative diagnosis of PCa is achieved through the acquisition of pathological results via needle biopsy—a benchmark procedure. Yet, this invasive method’s detection rate stands at a modest 62.2% [4]. The Gleason scoring system remains the cornerstone for assessing PCa’s malignancy degree [12–14]. Its utility extends to gauging biological activity, invasiveness, guiding treatment strategies, and prognostic evaluations. Furthermore, it emerges as a robust predictor of postoperative progression and survival [15]. The GS encompasses the summation of primary and secondary architectural patterns observed in hematoxylin and eosin-stained slides. A higher GS value denotes increased malignancy and invasiveness. Typically, GS ≤ 6 signifies a favorable prognosis and low risk, prompting active surveillance and vigilant monitoring instead of immediate intervention. GS = 7 represents an intermediate risk, often managed with monotherapy. Meanwhile, GS > 7 signifies high risk, warranting aggressive combined therapeutic strategies anchored in radical resection.

Notably, the distribution of Gleason patterns 3 and 4 exerts a bearing on patient prognoses. Consequently, GS = 7 PCa is parsed into two strata: GS 3 + 4 and GS 4 + 3. Evidence indicates diminished overall and specific survival rates for GS 4 + 3 in comparison to GS 3 + 4 [16]. Under specific circumstances like stable PSA levels and limited biopsy tumor volumes, guidelines even advocate for active monitoring among GS 3 + 4 patients [17]. Responding to this, a novel grading paradigm was unveiled in 2014, wherein GS = 7 is no longer treated as a single entity, but bifurcated into GS 3 + 4 (prognostic grade group II) and GS 4 + 3 (prognostic grade group III) [13]. To prevent undue biopsies and treatment in prostate patients, this study chiefly explores the feasibility of employing radiological imaging techniques to distinguish between benign, GS 3 + 3, GS 3 + 4, and GS 4 + 3 lesions.

It should be noted that previous studies often combined biopsy results with resection results; however, some studies [18, 19] have highlighted limitations in biopsies and tumor heterogeneity, leading to potential upgrading of pathological scores after PCa resection. To address this issue comprehensively, our study exclusively relied on radical prostatectomy pathology for all PCa lesions.

PCa lesions generally exhibit indistinct boundaries and irregular shapes, presenting as low signals in the PZ or TZ on T_2_WI. However, these visual attributes lack specificity, as there exists considerable overlap with images depicting prostate hyperplasia and inflammation. Leveraging the principles of DWI, which captures water diffusion dynamics, yields insights into tissue functionality at the cellular level. The resulting ADC map functions as a model delineating signal decay. Computed from multiple DWI *b*-values, this metric proves efficacious in unveiling lesions that elude detection through T_2_WI and standard DWI. Consequently, it demonstrates enhanced accuracy in localizing and detecting PCa [20]. Given that tumor tissues exhibit an augmented cellular composition, water molecule diffusion is impeded, consequently resulting in a distinct reduction in ADC values. The quantification of these ADC values serves as an objective measure of the degree of restricted water diffusion within the tissue [21].

This study’s outcomes manifest a significant diminution in both the median ADC_min_ and ADC_mean_ values for GS 4 + 3 when contrasted with GS 3 + 4. Additionally, the findings affirm heightened sensitivity and specificity when deploying ADC_min_ and ADC_mean_ for differentiating various low to intermediate-risk PCa grades. The inverse correlation observed between GS values and ADC_min_/ADC_mean_ substantiates the assertion that as the malignancy of prostate lesions intensifies, ADC values correspondingly decline, potentially portending an unfavorable PCa prognosis. Remarkably, these conclusions are aligned with the investigations of Saito et al [22] and Bajgiran et al [23]. Within the context of lesions spanning diverse low to intermediate-risk PCa grades, both ADC_min_ and ADC_mean_ exhibit diagnostic value, facilitating the nuanced prediction of risk stratification pertinent to prognosis. This capacity extends further, enabling the differentiation of higher-invasiveness PCa from an imaging perspective.

Upon juxtaposing the diagnostic performance of ADC_min_ and ADC_mean_, it becomes evident that ADC_mean_ consistently outperforms ADC_min_ in terms of sensitivity, specificity, the Youden index, and the AUC for low to intermediate-risk PCa diagnosis. This emphasis underscores the heightened discriminating power of ADC_mean_ in delineating PCa instances, corroborating the insights drawn from the work of Yang et al [24]. This phenomenon is potentially grounded in the heterogeneous composition of PCa tissue [25]. While ADC_min_ emphasizes regions characterized by the most prolific cellular proliferation and the densest distribution within tumor tissue—portraying the most potent constituents—ADC_mean_ reflects the holistic composition of the lesion. Moreover, considering that the GS amalgamates primary and secondary architectural patterns within cancerous tissue, it is postulated that ADC_mean_ encapsulates the lesion’s comprehensive nature most effectively, warranting a recommendation for radiologists to prioritize the integration of ADC_mean_ in their clinical practice.

This study is subject to several limitations. First, this study is a retrospective clinical study, which may entail some selection bias. Second, pathological results for benign patients were obtained through needle biopsy, which might involve a certain risk of missed diagnosis. Furthermore, the size of the selected ROI could affect the measurement of ADC values, thereby introducing some degree of error. Finally, this study is a single-center retrospective study, and whether the derived ADC threshold is applicable to other models requires further multicenter research.

In conclusion, a clear negative correlation between ADC values and PC’s invasiveness is evident. This inverse relationship suggests that as ADC values decline, the probability of malignancy in prostate lesions rises. ADC values exhibit remarkable sensitivity and specificity in diagnosing low to intermediate-risk PCa. Notably, ADC_mean_ proves more valuable than ADC_min_ in diagnosing low to intermediate-risk PCa. The diagnostic potential of ADC values is notably heightened for patients with G = 7, offering a robust tool for accurate preoperative assessment of tumor invasiveness. This, in turn, aids clinicians in devising tailored treatment strategies and making informed prognosis assessments. Furthermore, it serves as a means to prevent unnecessary biopsies and treatments. Additionally, radiologists are encouraged to prioritize the integration of ADC_mean_ for enhanced diagnostic precision. However, it’s essential to acknowledge the aforementioned limitations when interpreting the study’s outcomes.

### Supplementary Information

Electronic Supplementary Material

## Data Availability

The datasets used and analyzed during the current study are available from the corresponding author on reasonable request.

## Abbreviations

ADC: Apparent diffusion coefficient
ADC_mean_: Mean apparent diffusion coefficient
ADC_min_: Minimum apparent diffusion coefficient
AUC: Area under the curve
BL: Benign lesions
DWI: Diffusion-weighted imaging
GS: Gleason score
mpMRI: Multiparametric magnetic resonance imaging
PCa: Prostate cancer
PI-RADS V2.1: Prostate imaging and reporting data system version 2.1
PSA: Prostate-specific antigen
PZ: Peripheral zone
ROC: Receiver operating characteristic
ROI: Regions of interest
T_1_WI: T_1_-weighted imaging
T_2_WI: T_2_-weighted imaging
TZ: Transitional zone
