# Supplementary material for: The value of apparent diffusion coefficient values in predicting Gleason grading of low to intermediate-risk prostate cancer
Source: Insights Imaging. 2024 Jun 9;15:137. doi: 10.1186/s13244-024-01684-x (PMC11162992; doi:10.1186/s13244-024-01684-x)
Supplement: Supplementary file 1 — Electronic Supplementary Material [file 13244_2024_1684_MOESM1_ESM.pdf]

**The value of apparent diffusion coefficient values in predicting Gleason grading of low to intermediate-risk prostate cancer**

**ELECTRONIC SUPPLEMENTARY MATERIAL**

**Supplementary Table 1** Dispersion of lesions across different Gleason grading levels in various prostate zones

| Group | Number | Ratio (%) | Age (years) |
|---|---|---|---|
| **PZ** | 729 | 56.29 | 42~86 |
| BL | 192 | 26.34 | 45~85 |
| PCa | 537 | 73.66 | 42~85 |
| GS 3+3 | 107 | 14.68 | 53~86 |
| GS 3+4 | 276 | 37.86 | 42~85 |
| GS 4+3 | 154 | 21.12 | 49~85 |
| **TZ** | 566 | 43.71 | 49~91 |
| BL | 313 | 55.30 | 49~91 |
| PCa | 253 | 44.70 | 49~86 |
| GS 3+3 | 58 | 10.25 | 49~86 |
| GS 3+4 | 152 | 26.85 | 52~85 |
| GS 4+3 | 43 | 7.60 | 60~80 |

PZ, peripheral zone; TZ, transitional zone; BL, benign lesion; PCa, prostate cancer; GS, Gleason score.

**Supplementary Table 2** Distribution of median ADC values across various groups within different prostate zones

| Group | Number (%) | $ADC_{min}$ (×$10^{-3}$ $mm^2/s$, b = 800) | $ADC_{mean}$ (×$10^{-3}$ $mm^2/s$, b = 800) | Z value | P value |
|---|---|---|---|---|---|
| PZ BL | 192 (26.34) | 0.939 (0.822, 1.060) | 1.114 (1.003, 1.221) | -9.098 | < 0.001 |
| PZ PCa | 537 (73.66) | 0.514 (0.383, 0.665) | 0.803 (0.698, 0.891) | -20.760 | < 0.001 |
| TZ BL | 313 (55.30) | 0.837 (0.740, 0.925) | 1.031 (0.958, 1.100) | -16.028 | < 0.001 |
| TZ PCa | 253 (44.70) | 0.494 (0.392, 0.606) | 0.792 (0.721, 0.864) | -16.487 | < 0.001 |

PZ, peripheral zone; TZ, transitional zone; BL, benign lesion; PCa, prostate cancer; ADC, apparent diffusion coefficient; $ADC_{min}$, minimum apparent diffusion coefficient; $ADC_{mean}$, mean apparent diffusion coefficient; GS, Gleason score.
